# Fabricating an Aqueous Symmetric Supercapacitor with a Stable High Working Voltage of 2 V by Using an Alkaline–Acidic Electrolyte

**DOI:** 10.1002/advs.201801665

**Published:** 2018-11-08

**Authors:** Chunyang Li, Wenzhuo Wu, Peng Wang, Weibin Zhou, Jing Wang, Yuhui Chen, Lijun Fu, Yusong Zhu, Yuping Wu, Wei Huang

**Affiliations:** ^1^ State Key Laboratory of Materials‐oriented Chemical Engineering Nanjing Tech University Nanjing 211816 China; ^2^ Institute of Advanced Materials (IAM) Nanjing Tech University Nanjing 210009 China; ^3^ Guanghua Cambridge International School Shanghai 201315 China; ^4^ School of Energy Science and Engineering Nanjing Tech University Nanjing 211816 China

**Keywords:** activated carbon, alkaline–acidic electrolytes, aqueous, high voltages, symmetric supercapacitors

## Abstract

Aqueous symmetric carbon‐based supercapacitors (CSCs) are always the research focus for energy storage devices because of the virtue of low cost, inherent safety, and encouraging electrochemical stability. As is well‐known, so far most aqueous symmetric CSCs are subjected to low energy densities. Here, a symmetric supercapacitor comprising electrodes from biomass‐derived activated carbon and alkaline–acidic electrolyte is reported. This aqueous symmetric CSC demonstrates exceptional electrochemical performance with high stable working voltage of 2 V and attractive cycling stability of no capacitance loss over 10 000 cycles. Impressively, it shows a remarkable energy density of 36.9 W h kg^−1^ at 248 W kg^−1^ based on the total mass of the active materials, which is much higher than traditional aqueous symmetric CSCs, and a power density of 4083 W kg^−1^ with an energy density of 8.8 W h kg^−1^. The use of stable alkaline–acidic electrolyte provides an innovative technique to enhance the energy density of aqueous supercapacitors.

The expanding demand for portable storage devices, electric vehicles, and smart grids has hastened the growth of all‐solid‐state supercapacitors and batteries with low price, high voltage, and durability.^1^, ^2^, ^3^, ^4^ Unlike batteries, supercapacitors, also viewed as electrochemical capacitors, hold and release the electrochemical energy by electrical double layer (EDL) capacitance or pseudocapacitance, featuring high power density, fast kinetic reactions, and longevity (over 10^6^ cycles), which have brought about promising applications for meeting current power needs.^5^, ^6^, ^7^ There are commonly two categories of supercapacitors on basis of their electrolytes: aqueous supercapacitors and nonaqueous organic supercapacitors.^8^, ^9^ Due to high ionic conductivity of aqueous electrolytes, aqueous supercapacitors possess much higher power density than nonaqueous organic ones. In addition, the assembly of aqueous supercapacitors does not need anhydrous condition.^10^, ^11^, ^12^ However, aqueous supercapacitors demonstrate lower energy density relative to nonaqueous organic ones, especially carbon‐based supercapacitors (CSCs), as a result of the restriction of water decomposition, whose theoretic voltage is 1.229 V.^13^, ^14^, ^15^

The energy densities of supercapacitors are reliant on both operating voltage and specific capacitance according to *E*  = 1/2 *CV* ^2^. Limited by water splitting, aqueous symmetric CSCs deliver a narrow cell voltage range (0–1 V) in common electrolytes such as H_2_SO_4_ or KOH, engendering low energy density (<10 W h kg^−1^).^16^, ^17^, ^18^ For the sake of conquering the above problem, some researchers have exploited symmetric CSCs with neutral electrolyte including Li_2_SO_4_, LiNO_3_, Na_2_SO_4_, NaNO_3_ etc., exhibiting higher working voltages.^19^, ^20^, ^21^, ^22^, ^23^ Gao et al. investigated the cell voltage of symmetric supercapacitors (SSCs) based on H_2_O_2_‐treated commercial carbon in 2 m Li_2_SO_4_. A maximum working voltage of 1.9 V was attained with an outstanding cycling performance.^24^ Porous 3D interconnected carbon framework‐based SSCs, working at a stable voltage of 1.6 V, showed excellent cycling behavior in 1 m Na_2_SO_4_ with no capacitance loss after 3000 cycles reported by Bello et al.^25^ Such voltage increments of aqueous symmetric CSCs are ascribed to high dihydrogen overpotentials in negative electrode.^19^, ^24^ Recently, Yu et al. precharged multiscale porous carbon electrodes with a constant potential to tune its surface charge, which expands the cell voltage of SSCs from 1.4 to 1.8 V and leads to a double increase of energy density compared to the raw SSCs.^26^ Symmetric CSCs with above electrolytes, however, have also low specific energy due to its low capacitance resulted from lower conductivity of neutral electrolytes in comparison with H_2_SO_4_ and KOH.^7^, ^27^

Nowadays, almost all commercial supercapacitors use carbons as electrode materials instead of pseudocapacitance materials with high specific capacitance, since carbon materials have low cost, environmental benignity, high electrical conductivity, as well as excellent cycling stability.^8^, ^28^, ^29^, ^30^, ^31^ Among several carbon materials, activated carbons with abundant sources dominate the electrode materials for EDL capacitors because of their significant cost advantage, numerous porous structure, and self‐doped heteroatom, originating from biomass such as dead plants, animal carcasses, and life wastes.^32^, ^33^, ^34^, ^35^ Longan (Dimocarpus longan Lour) is a nutrient‐rich fruit, and called “Gui Yuan” in China when it is processed into dried pulp, which is mainly distributed in China's coastal areas. However, hundreds of tons of longan seeds (LS) are abandoned, generating a huge waste of biomass every year, even though LS can be served as the raw material for extracting phenolic compounds.^36^, ^37^ Considering the rich amino acids in LS, it should be an excellent carbon precursor for transforming into biomass‐derived activated carbon.

Herein, we demonstrate that the conjunction of alkali and acid electrolyte containing potassium ion (denoted as alkaline–acidic electrolyte) via a K^+^‐conducting Nafion membrane promotes the cell voltage of CSC with KOH‐activated biomass‐derived carbons. The Longan seed‐derived activated carbon (LS AC) presents the maximum capacitances of 307 F g^−1^ in 1 m H_2_SO_4_ (0.5 A g^−1^) and 260 F g^−1^ in 2 m KOH (0.5 A g^−1^), respectively, and outstanding electrochemical stability. The alkaline–acidic electrolyte combines the low H_2_ evolution potential (2 m KOH solution) for the negative electrode with the high O_2_ evolution potential (1 m H_2_SO_4_ solution) for the positive electrode, so that the symmetric supercapacitor consisting of LS AC electrodes and the alkaline–acidic electrolyte realizes a high stable working voltage of 2 V. The assembled symmetric supercapacitor exhibits excellent cycling stability and tremendous advance in energy density of 36.9 W h kg^−1^ at 248 W kg^−1^ based on the total mass of active materials.

The longan seed‐derived activated carbons were manufactured by a simple two‐step method: carbonization and KOH activation. The products originated from three mass ratios of carbon precursor and KOH (1:1, 1:3, and 1:5) are marked as LS AC 1–1, LS AC 1–3, and LS AC 1–5, respectively. The microstructures of the three samples were studied via scanning electron microscopy (SEM) and transmission electron microscopy (TEM), as depicted in **Figure** **1**. All samples of LS ACs manifest irregular microblocks and unevenly distributed cavities regardless of the contents of KOH activation agent. Besides, plentiful pores exist in all samples of LS ACs, which are mostly derived from the KOH activation. From the TEM images of LS ACs, it can be seen that the pore distribution and roughness of the surface of samples change with the increase of KOH contents. LS AC 1–1 shows a smooth surface with graphene‐like layers and pores (Figure 1d). When the content of KOH in activation process is improved to 83.3%, LS AC 1–5 demonstrates a rough surface with multitudinous pores (Figure 1f). Besides, the surface chemical compositions of all LS AC samples are determined by energy dispersive X‐ray (EDX) analysis (Figure S1 and Table S1, Supporting Information). It is clear that all LS AC samples are composed of C, N, and O elements, and show high N content of 9.51 wt% for LS AC 1–1, 7.76 wt% for LS AC 1–3, and 6.25 wt% for LS AC 1–5, which could be relevant to the KOH activation.

**Figure 1:**
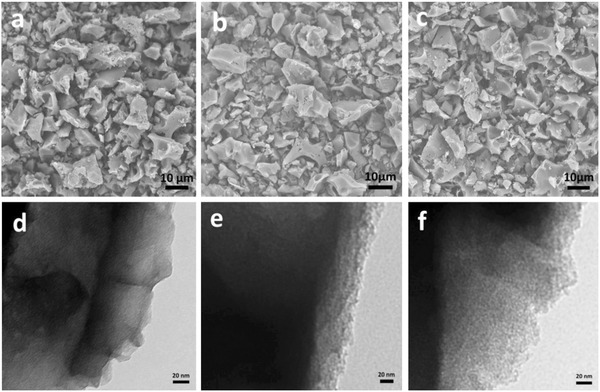
SEM and TEM images of LS AC sample: a,d) LS AC 1–1, b,e) LS AC 1–3, c,f) LS AC 1–5.

Two broad characteristic peaks at round 22.5°and 43°can be observed from the X‐ray diffraction (XRD) patterns of all samples in **Figure** **2**a, verifying the amorphous structure of LS ACs.^38^, ^39^ There are clear shifts of (002) peaks involving angles and intensities, corresponding to various contents of N and O heteroatoms stemmed from different activation degree.^14^ All samples display similar (100) characteristic peaks at around 43°, demonstrating the limited graphitization degree of LS AC.^32^ The Raman spectra further testifies the amorphous nature of LS ACs by the characteristic peaks (Figure 2b): D band at about 1350 cm^−1^ indicating the disordered and defective carbon structures, and G band at around 1590 cm^−1^ representing the vibration of the carbon atoms. The intensity ratio of D and G band (*I*_D_/*I*_G_) reflects the graphitic order of LS ACs.^40^, ^41^ The values of *I*_D_/*I*_G_ of all samples are close to 1.00, unrelated with the KOH ratios, suggesting the highly disordered characteristic of LS ACs with low graphitization degree.

**Figure 2:**
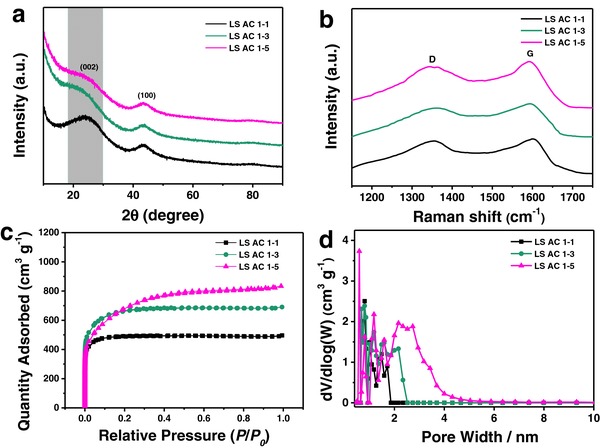
a) XRD patterns, b) Raman spectra, c) N_2_ absorption–desorption isotherm curves, and d) pore size distribution of LS AC 1–1, LS AC 1–3, and LS AC 1–5.

The N_2_ absorption–desorption isotherm curves of all samples at 77 K (Figure 2c) belong to typical type‐I based on the International Union of Pure and Applied Chemistry (IUPAC) classification, indicating the microporous structure of KOH‐activated carbon. It is clear that the sharp slopes appear in isotherms at the low relative pressure from LS AC 1–1 to LS AC 1–5, confirming the existence of abundant micropores and a few mesopores. From the pore size distributions (0.4–10 nm, density functional theory (DFT) model) of LS AC 1–1, LS AC 1–3, and LS AC 1–5 (Figure 2d), all samples have large quantities of micropores. Small‐sized mesopores in the range of 2–4 nm emerge particularly in LS AC 1–5, which is ascribed to KOH activation with different ratios.^42^ Table S2 (Supporting Information) compares the main physical performance of all samples. LS AC 1–5 possesses the highest Brunauer–Emmet–Teller (BET) specific surface area of 2325 m^2^ g^−1^ and total pore volume, which are beneficial to present the better capacitance and rate properties.^28^

The capacitive properties of all LS ACs were examined using three‐electrode configuration in 1 m H_2_SO_4_ and 2 m KOH aqueous electrolyte solution, respectively. First, the quasirectangular cyclic voltammetry (CV) curves at 10 mV s^−1^ and symmetric galvanostatic charge–discharge (GCD) curves at 1 A g^−1^ of all LS AC electrodes in 1 m H_2_SO_4_ aqueous electrolyte solution (the potential range from 0 to 1 V vs saturated calomel electrode (SCE)) are depicted in **Figure** **3**a,b, respectively, indicating the excellent coulombic efficiency and electrical double‐layer capacitive behavior with small redox peaks from the functional groups such as C=O.^43^, ^44^ The discharge specific capacitances are 237 F g^−1^ for LS AC 1–1, 265 F g^−1^ for LS AC 1–3, and 285 F g^−1^ for LS AC 1–5 at the current density of 1 A g^−1^ (calculated from Figure 3b). Obviously, LS AC 1–5 electrode shows the best capacitive performance, which is attributed to the higher specific surface area and total pore volume relative to other LS AC samples. Figure 3c displays the CV curves of LS AC 1–5 electrode at different scan rates. As the scan rate is increased to 100 mV s^−1^, the CV curve still maintains quasirectangular shape, suggesting an excellent rate performance resulted from the mesoporous and microporous nature. The GCD profiles of all LS AC electrodes at various current densities ranging from 0.5 to 30 A g^−1^ are displayed in Figure 3d,e and Figure S2 (Supporting Information). The discharge specific capacitances of LS AC 1–5 are 307, 285, 253, 230, 217, 210, 204, 189, and 181 F g^−1^ at 0.5, 1, 2, 4, 6, 8, 10, 20, and 30 A g^−1^ (Figure 3f), respectively, indicating remarkable rate capability with 58.9% capacitance retention, superior to other LS AC samples (48% for LS AC 1–3 and 42% for LS AC 1–1), which could be caused by the high mesopore volume of LS AC 1–5. From the electrochemical impedance spectroscopy (Figure 3g) of LS AC 1–5, there is a low charge transfer resistance (*R*_CT_) of ≈0.8 Ω, manifesting the reaction of fast ion diffusion. The cycling performance of LS AC 1–5 electrode is evaluated by continuous galvanostatic charge–discharge test at a current density of 5 A g^−1^. As shown in Figure 3h, LS AC 1–5 delivers an outstanding electrochemical stability with 96.5% capacitance retention after 10 000 cycles.

**Figure 3:**
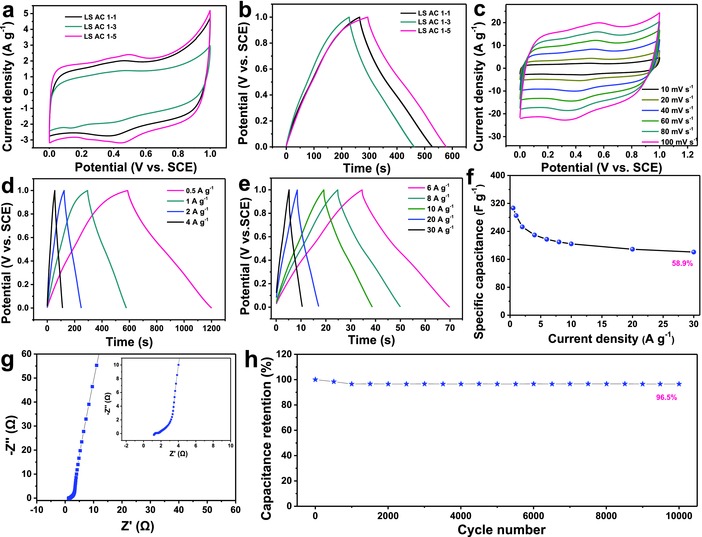
Electrochemical performance of LS AC electrodes in the 1 m H_2_SO_4_ aqueous solution: a) CV curves at 10 mV s^−1^ and b) GCD profiles at 1 A g^−1^ of all LS AC electrodes. c) CV curves of LS AC 1–5 electrode at different scan rates. d,e) GCD profiles of LS AC 1–5 electrode at various current densities. f) Specific capacitance versus current density of LS AC 1–5 electrode. g) Nyquist plot of LS AC 1–5 electrode (inset is enlarged spectra). h) Cycling stability of LS AC 1–5 electrode at 5 A g^−1^ over 10 000 cycles.

The CV curves of all LS AC electrodes in the potential window between −1 and 0 V (vs Hg/HgO) also display quasirectangular shape at the scan rate of 10 mV s^−1^ in 2 m KOH aqueous electrolyte solution (**Figure** **4**a). Calculated from Figure 4b, the discharge specific capacitances are 174 F g^−1^ for LS AC 1–1, 187 F g^−1^ for LS AC 1–3, and 260 F g^−1^ for LS AC 1–5, respectively, at the current density of 0.5 A g^−1^. Similar to the behaviors in 1 m H_2_SO_4_ aqueous electrolyte solution, LS AC 1–5 also shows the preferable capacitive performance. The CV curves of LS AC 1–5 electrode at various scan rates in Figure 4c also hold quasirectangular and symmetric shape without a conspicuous deformation, demonstrating the characteristic of EDL capacitance and noteworthy rate capability. Figure 4d,e and Figure S3 (Supporting Information) reveal the GCD profiles of all LS AC electrodes at diverse current densities. In accordance with the consequence of CV curves, LS AC 1–5 exhibits an exceptional rate performance with 59.2% retention as the current densities increase from 0.5 to 20 A g^−1^ (Figure 4f). LS AC 1–5 shows a sharp slope and small diameter of semicircle (≈1.8 Ω), suggesting the low *R*_CT_ and quick reaction kinetics (Figure 4g). Impressively, LS AC 1–5 shows an excellent cycling stability with only 2% capacitance fading after 10 000 cycles at a current density of 3 A g^−1^ (Figure 4h).

**Figure 4:**
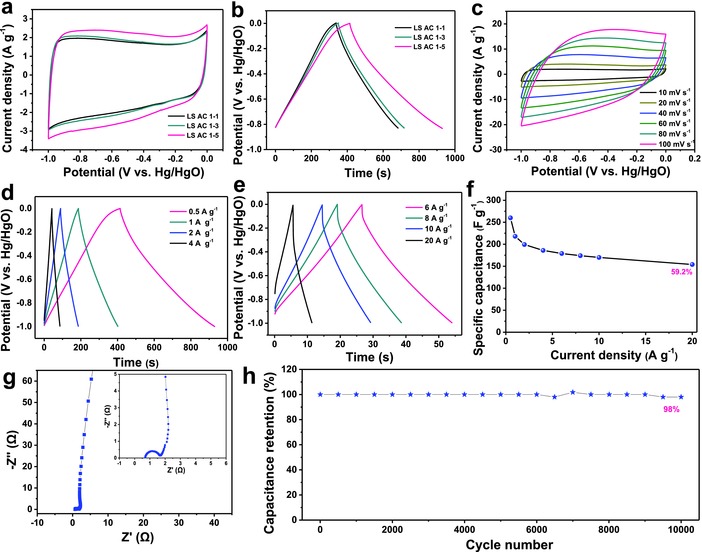
Electrochemical performance of LS AC electrodes in the 2 m KOH aqueous solution: a) CV curves at 10 mV s^−1^ and b) GCD profiles at 0.5 A g^−1^ of all LS AC electrodes. c) CV curves of LS AC 1–5 electrode at different scan rates. d,e) GCD profiles of LS AC 1–5 electrode at various current densities. f) Specific capacitance versus current density of LS AC 1–5 electrode. g) Nyquist plot of LS AC 1–5 electrode (inset is enlarged spectra). h) Cycling stability of LS AC 1–5 electrode at 3 A g^−1^ over 10 000 cycles.

To achieve high operating voltage and energy density at the same time, a special alkaline–acidic electrolyte: 2 m KOH (0.25 m K_2_SO_4_) and 1 m H_2_SO_4_ (0.25 m K_2_SO_4_) separated by K^+^‐conducting Nafion membrane is fabricated as the electrolyte for symmetric supercapacitor with high‐capacitance KOH‐activated biomass carbon. In theory, by the combination of the low H_2_ evolution potential from KOH solution in the negative electrode (−0.829 V vs standard hydrogen electrode (SHE)) with the high O_2_ evolution potential from H_2_SO_4_ solution in the positive electrode (1.229 V vs SHE), a high stable working voltage beyond 2 V (much above 1.23 V) could be obtained (Figure S4, Supporting Information). It is mentioned above that the stable potential windows are 0–1 V (vs SCE, 0.241–1.241 V vs SHE) and −1–0 V (vs Hg/HgO, −0.902–0.098 vs SHE) in 1.0 m H_2_SO_4_ and 0.5 m KOH aqueous electrolyte solution, respectively (Figure S5, Supporting Information). Therefore, the potential window voltage of symmetric supercapacitor based on LS AC 1–5 can be stable at 2 V without any obvious gas evolution in the alkaline–acidic electrolyte. It is essential for LS AC based supercapacitor to balance the charges between positive and negative electrodes to achieve high energy density.^45^, ^46^ Based on the equation of charge stored at each electrode, *q* = *I* × *t* = *C*_s_ × Δ*V* × *m*, where *I* is the charge (discharge) current (A), *t* is the discharge time (s), *C*_s_ is the specific capacitance (F g^−1^), Δ*V* is the potential window during discharging (V), and *m* is the mass of active electrode material (g), the mass ratio of positive and negative electrodes should follow this equation,$m_{+}/m_{-}=(C_{s}^{-}\;\times \;\Delta V_{-})/(\;C_{s}^{+}\times \;\Delta V_{+})$.^47^, ^48^ According to above results, the ideal weight ratio of positive and negative electrodes is 1:1.3, and the mass of active electrode material of LS AC based supercapacitor contains the total mass of two active electrode materials.

The CV curves of LS AC based supercapacitor demonstrate an ideal charge–discharge behavior of EDL capacitance and rate performance from 0 to 2 V at different scan rates of 5–80 mV s^−1^, which is consistent with previous opinion (**Figure** **5**a). The GCD profiles of LS AC based supercapacitor from 0.25 to 6 A g^−1^ are nearly symmetric (Figure 5b,c) and further indicate the EDL feature and good rate capability with 50.7% capacitance retention when the current density is increased from 0.25 to 6 A g^−1^. The LS AC based supercapacitor on basis of alkaline–acidic electrolyte delivers a high discharge specific capacitance of 52.6 F g^−1^ at a current density of 1 A g^−1^. To assess the cycling stability of LS AC based supercapacitor using alkaline–acidic electrolyte, a repeated galvanostatic charge–discharge test is performed at a current density of 5 A g^−1^ (Figure 5e). The LS AC based supercapacitor presents an exceptional electrochemical stability with no capacitance loss after 10 000 cycles and low *R*_CT_ of ≈20 Ω (Figure 5f). During 4000 cycles, the LS AC based supercapacitor undergoes an activation process and stabilizes. After 4000 cycles, a slow decay of capacitance appears, which could be assigned to the degradation of K^+^‐conducting Nafion membrane from OH^−^ attack.^49^ The LS AC based supercapacitors using 2 m KOH, 1 m H_2_SO_4_, and 1 m Li_2_SO_4_ aqueous electrolyte solutions also were investigated (Figures S6–S8, Supporting Information). It can be seen that 1 m Li_2_SO_4_ aqueous electrolyte solution indicates a higher internal resistance of 51.7 Ω and *R*_CT_ of ≈24 Ω relative to other two supercapacitors, agreeing with previously reported results (Figure 5f).^27^, ^50^ The energy and power densities are the crucial parameters for evaluating the practical application of supercapacitors.^51^, ^52^ Calculated from the GCD profiles of LS AC based supercapacitor at different current densities and electrolytes, Ragone plots are drawn to analyze the energy densities of LS AC based supercapacitors (Figure 5g). Impressively, the LS AC based supercapacitor using alkaline–acidic electrolyte reveals a much higher energy density of 36.9 W h kg^−1^ at a power density of 248 W kg^−1^ compared with the conventional symmetric supercapacitors using 2 m KOH, 1 m H_2_SO_4_, and 1 m Li_2_SO_4_ aqueous electrolyte solutions (Figure 5g), which outperform previous aqueous symmetric CSCs such as ZIF‐8 derived carbon (10.8 W h kg^−1^ at 225 W kg^−1^, 0–0.9 V),^53^ lotus seedpods derived carbon (12.5 W h kg^−1^ at 260 W kg^−1^, 0–1 V),^13^ 3D flower‐like carbon (15.9 W h kg^−1^ at 317.5 W kg^−1^, 0–1.8 V),^54^ and carbon nanofibers‐graphene sheets (29 W h kg^−1^ at 450 W kg^−1^, 0–1.8 V).^21^ Moreover, an energy density of 8.8 W h kg^−1^ still can be obtained at higher power density of 4 kW kg^−1^. Such high energy density for aqueous SSCs based on activated carbon can be assigned to the optimal use of H_2_/O_2_ evolution overpotentials from the alkaline–acidic electrolyte as well as the capacitance of LS ACs. Self‐discharge is a common phenomenon for supercapacitors, which can be investigated by monitoring the stability of open circuit voltage (OCV). LS AC based supercapacitors in 2 m KOH, 1 m H_2_SO_4_, 1 m Li_2_SO_4_, and alkaline–acidic electrolytes were fully charged to 1, 1, 1.6, and 2 V at 1 A g^−1^, respectively. Within 8 h, a relatively stable OCV of LS AC based supercapacitor in alkaline–acidic electrolyte indicates a smaller self‐discharge (Figure S9, Supporting Information). A green (2.4 V) light‐emitting diode (LED) is lit by two simple cells, indicating the practicability of LS AC based supercapacitor using alkaline–acidic electrolyte (Figure S10, Supporting Information). Therefore, the stable alkaline–acidic electrolyte has great promising application for improving the energy densities of aqueous symmetric CSCs. Its power density can be up to 4083 W kg^−1^ with an energy density of 8.8 W h kg^−1^. In addition, it is also suitable for high‐capacitance carbon materials and some high‐performance corrosion‐resisted metal oxide materials.

**Figure 5:**
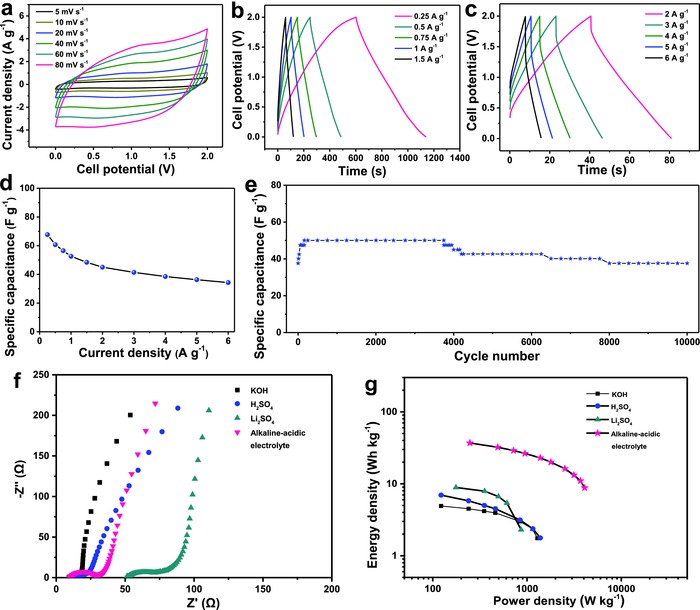
Electrochemical performance of LS AC based supercapacitor in alkaline–acidic electrolyte: a) CV curves at different scan rates. b,c) GCD profiles at various current densities. d) Specific capacitance versus current density. e) Cycling stability at 5 A g^−1^ over 10 000 cycles. f) Nyquist plots and g) Ragone plots compared with LS AC based supercapacitors in 2 m KOH, 1 m H_2_SO_4_, and 1 m Li_2_SO_4_ aqueous solution.

In summary, we report a novel and stable alkaline–acidic electrolyte to fabricate high‐voltage and high‐energy symmetric supercapacitor with KOH‐activated biomass carbon. The as‐prepared LS AC 1–5 sample delivers high capacitance of 307 F g^−1^ in 1 m H_2_SO_4_ at 0.5 A g^−1^ and 260 F g^−1^ in 2 m KOH at 0.5 A g^−1^, respectively, and excellent cycling stability with negligible decay after 10 000 cycles. The as‐fabricated symmetric supercapacitor with alkaline–acidic electrolyte and LS AC 1–5 electrodes presents a high stable working voltage of 2 V, outstanding cycling performance with no capacitance fading after 10 000 cycles, and remarkable energy density of 36.9 W h kg^−1^ at a power density of 248 W kg^−1^ based on the total mass of two active electrode materials. Its power density can be up to 4083 W kg^−1^. Therefore, the construction of stable alkaline–acidic electrolyte brings a significant advance in energy density as a new approach for the future research of supercapacitors.

## Experimental Section

*Preparation of LS ACs*: Longan seeds were broken into small particles (about 1–2 mm) and washed with deionized (DI) water and ethanol, which were dried at 120 °C for 10 h to remove the excess water. The dried longan seeds were carbonized in tube furnace at 450 °C for 1.5 h in N_2_ atmosphere. The carbon precursor was milled into powder after naturally cooling to room temperature. The black powder and potassium hydroxide (KOH) were mixed at various weigh ratios of 1:1, 1:3, and 1:5 with a certain amount of DI water to form slurry. After drying at 80 °C, the mixtures were activated at 800 °C for 1.5 h in N_2_ atmosphere. The activated carbons were rinsed with 1 m HCl and DI water until it was neutral, then dried at 80 °C overnight.

*Preparation of K^+^‐Conducting Nafion Membrane*: The K^+^‐conducting Nafion membrane was prepared by treating the waste Nafion NR212 membrane from Yinfeng New Energy Co. Ltd. with the following three steps inspired by a latest work of Lu and co‐workers.^55^ First, Nafion membrane was soaked in 3 wt% H_2_O_2_ aqueous solution at 80 °C for 1 h. It was rinsed with DI water to remove the remaining H_2_O_2_. Second, the waste Nafion membrane was soaked in 1 m H_2_SO_4_ at 80 °C for 1 h and washed with DI water until pH reached 7. Finally, it was soaked in 1 m KCl aqueous solution (pH was adjusted to 1 by using HCl) at 60 °C for 12 h to covert it from the H^+^‐conducting to the K^+^‐conducting.

*Material Characterization*: The phase and structure of LS ACs were investigated by XRD (SmartLab TM 3KW, Cu Ka radiation) and Raman spectra (WITEC Alpha300M+). The morphologies of LS ACs were examined by SEM (Phenom ProX) and TEM (HITACHI HF3300). N_2_ adsorption–desorption measurements were performed by ASAP 2460 (Micrometritics, USA) at 77 K. The specific surface areas and pore size distributions were calculated by using the BET method and Barrett–Joyner–Halenda model.

*Electrochemical Measurements*: All electrochemical measurements apart from the cycling stability test were accomplished by a CHI 760E electrochemical workstation (Chenhua, Shanghai) at room temperature. The cycling performance was examined by a CT2001A cell tester (Land, Wuhan). The LS AC electrodes in 1 m H_2_SO_4_ solution were prepared by pressing a film comprising active material, acetylene black, and poly (tetrafluoroethylene) in a weight ratio of 8:1:1 onto carbon rod. The LS AC electrodes in 2 m KOH and 1 m Li_2_SO_4_ solution were fabricated in the same way as the electrode in H_2_SO_4_ solution onto Ni‐mesh. The electrochemical tests of individual electrodes were conducted by three‐electrode system with platinum wire electrode and SCE (1 m Li_2_SO_4_, 1 m H_2_SO_4_) or Hg/HgO (2 m KOH) as counter and reference electrodes, respectively. The CV and GCD measurements of LS AC based supercapacitors with two‐electrode configuration were recorded at 0–1 V in 1 m H_2_SO_4_ and 2 m KOH aqueous solution, 0–1.6 V in 1 m Li_2_SO_4_ aqueous electrolyte solution, and 0–2.0 V in alkaline–acidic electrolyte (2 m KOH (0.25 m K_2_SO_4_) and 1 m H_2_SO_4_ (0.25 m K_2_SO_4_) separated by K^+^‐conducting Nafion membrane). The discharge specific capacitances (*C*_s_) of single electrodes or supercapacitors were calculated from the GCD curves using the following equation, Equation (1):

(1) $$C_{s}=\frac{I\times t}{m\times \Delta V}\left(F\;g^{-1}\right)$$

where *I* is the discharge current (A), *t* is the discharge time (s), *C*_s_ is the specific capacitance (F g^−1^), Δ*V* is the discharge potential window excluding the IR drop (V), and *m* is the mass or total mass of active electrode material (g).

During the galvanostatic charge/discharge of supercapacitors, the energy density (*E*) and power density (*P*) were calculated by the following equations, Equations (2) and (3):

(2) $$E=\frac{C_{s}\times \Delta V^{2}}{2\times 3.6}\;\left(W\;h\;{\mathrm{kg}}^{-1}\right)$$

(3) $$P=\frac{E\times 3600}{t}\left(W\;{\mathrm{kg}}^{-1}\right)$$

## Conflict of Interest

The authors declare no conflict of interest.

## Supporting information

Supplementary
