# Supplementary material for: Fabricating an Aqueous Symmetric Supercapacitor with a Stable High Working Voltage of 2 V by Using an Alkaline–Acidic Electrolyte
Source: Adv Sci (Weinh). 2018 Nov 8;6(1):1801665. doi: 10.1002/advs.201801665 (PMC6325591; doi:10.1002/advs.201801665)
Supplement: Supplementary file 1 — Supplementary [file ADVS-6-1801665-s001.pdf]

Copyright WILEY-VCH Verlag GmbH & Co. KGaA, 69469 Weinheim, Germany, 2018.

Supporting Information

for *Adv. Sci.*, DOI: 10.1002/advs.201801665

Fabricating an Aqueous Symmetric Supercapacitor with a Stable High Working Voltage of 2 V by Using an Alkaline–Acidic Electrolyte

Chunyang Li, Wenzhuo Wu, Peng Wang, Weibin Zhou, Jing Wang, Yuhui Chen, Lijun Fu, Yusong Zhu,* Yuping Wu,* and Wei Huang*

Copyright WILEY-VCH Verlag GmbH & Co. KGaA, 69469 Weinheim, Germany, 2016.

Supporting Information

Fabricating aqueous symmetric supercapacitor with a stable high working voltage of 2 V by using an alkaline-acidic electrolyte

Chunyang Li, Wenzhuo Wu, Peng Wang, Weibin Zhou, Jing Wang, Yuhui Chen, Lijun Fu, Yusong Zhu*, Yuping Wu,* and Wei Huang*

C. Li, Prof. Y. Chen, Prof. L. Fu, Prof. Y. Wu
State Key Laboratory of Materials-oriented Chemical Engineering,
Nanjing Tech University
Nanjing 211816, China
E-mail: l.fu@njtech.edu.cn; wuyp@fudan.edu.cn
C. Li, P. Wang, W. Zhou, Prof. Y. Wu, Prof. W. Huang
Institute of Advanced Materials (IAM)
Nanjing Tech University
Nanjing 210009, China
W. Wu
Guanghua Cambridge International School, Shanghai 201315, China
Dr. J. Wang, Prof. Y. Chen, Prof. L. Fu, Dr. Y. Zhu, Prof. Y. Wu
School of Energy Science and Engineering
Nanjing Tech University
Nanjing 211816, China
E-mail: zhuys@njtech.edu.cn;

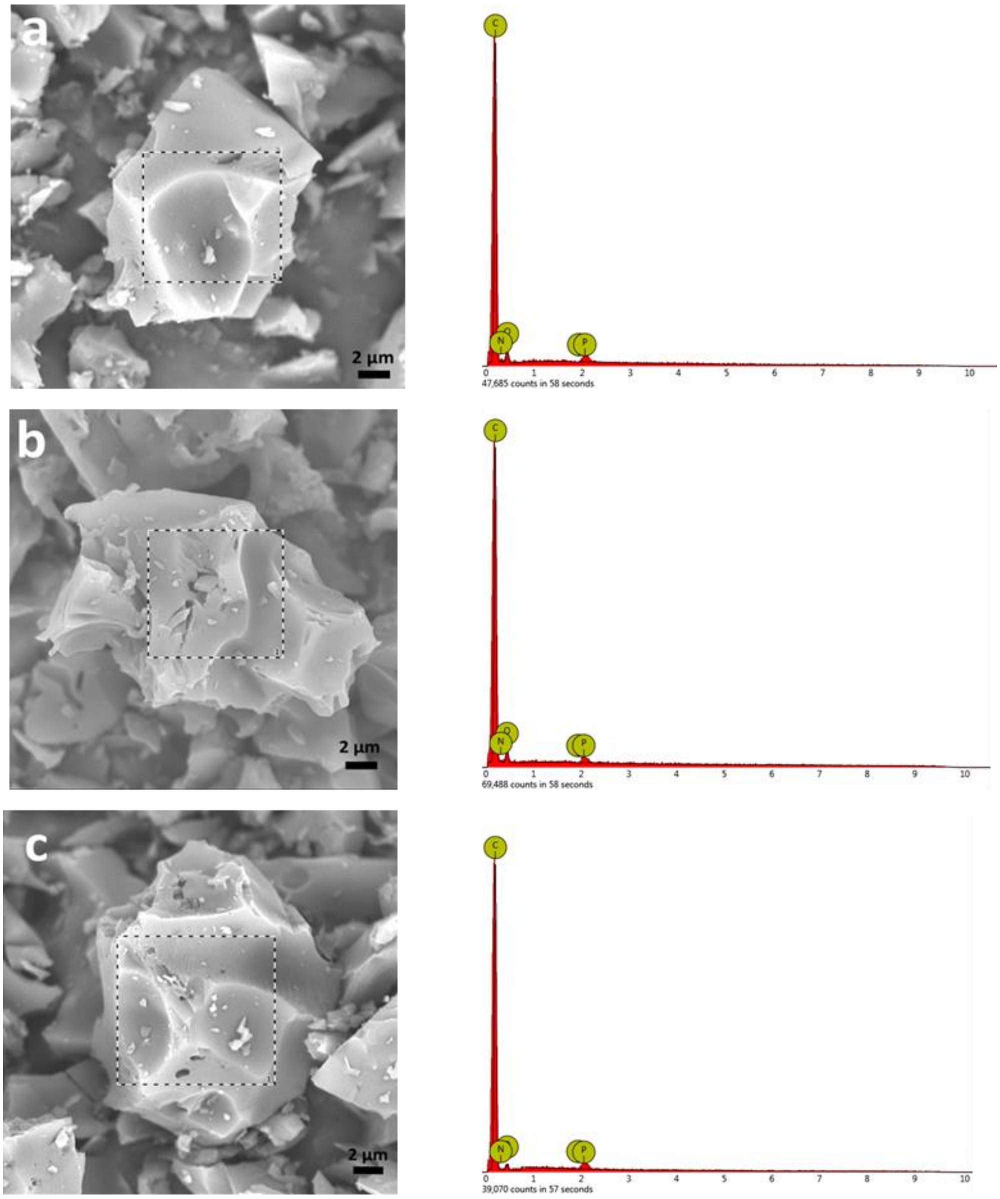

Figure S1. The EDX analysis: a) LS AC 1-1, b) LS AC 1-3, and c) LS AC 1-5.

Table S1. Surface chemical composition of all LS AC samples determined by EDS analysis.

Samples	C wt.% (At.%)	O wt.% (At.%)	N wt.% (At.%)
LS AC 1-1	79.22 (82.66)	11.27 (8.83)	9.51 (8.51)
LS AC 1-3	80.55 (83.93)	11.68 (9.14)	7.76 (6.94)
LS AC 1-5	86.87 (89.18)	6.27 (5.52)	6.87(5.30)

Table S2. Comparison of physical properties of LS ACs.

Samples	S_{BET} (m^2 g^{-1})	V_{total} (m^3 g^{-1})	V_{mic} (m^3 g^{-1})	Mean pore diameter (nm)	I_D/I_G
LS AC 1-1	1492	0.75	0.68	2.02	0.99
LS AC 1-3	2080	1.05	0.71	2.02	0.99
LS AC 1-5	2325	1.26	0.83	2.17	0.99

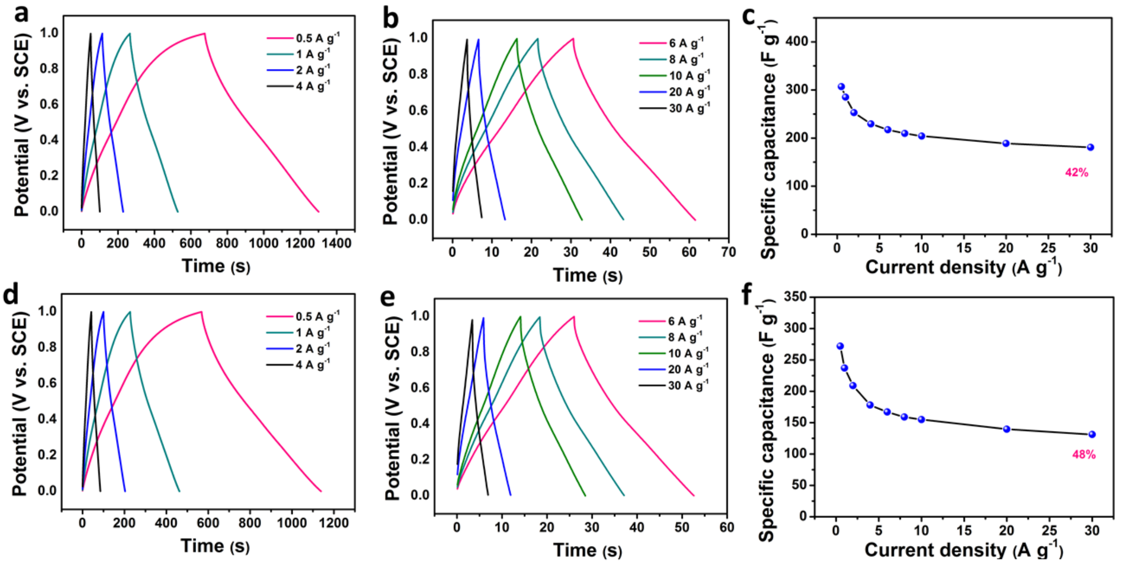

Figure S2. GCD profiles of LS AC 1-1 a,b) and LS AC 1-3 d,e) samples at current density of 0.5 to 30 A g^{-1} in 1 M H_2SO_4 aqueous electrolyte solution. Specific capacitance vs. current density of LS AC 1-1 c) and LS AC 1-3 f) samples.

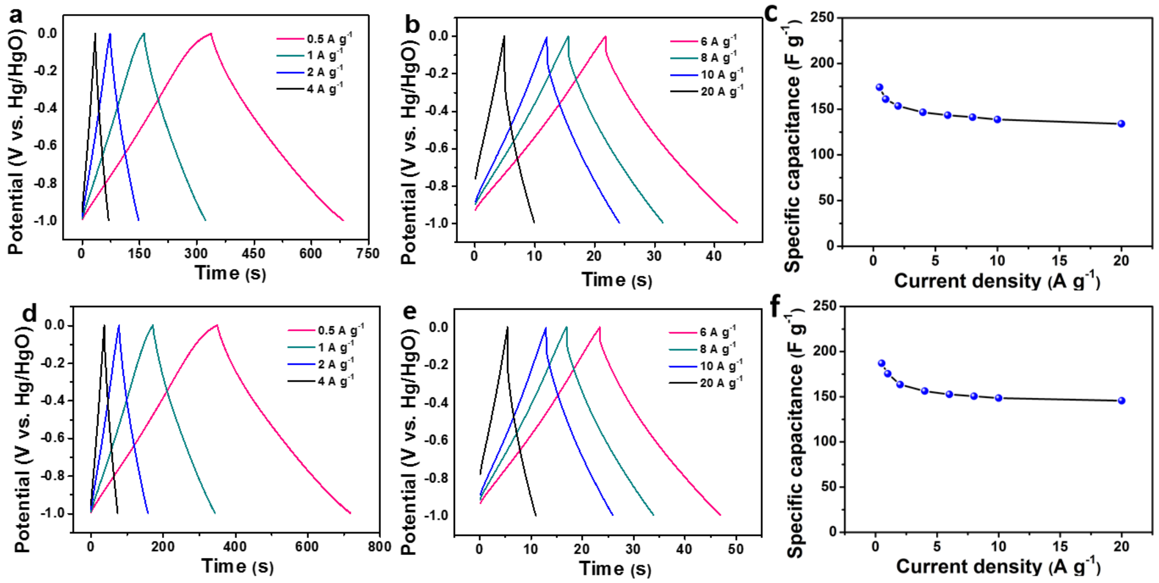

Figure S3. GCD profiles of LS AC 1-1 a,b) and LS AC 1-3 d,e) samples at current density of 0.5 to 20 A g^{-1} in 2 M KOH aqueous electrolyte solution. Specific capacitance vs. current density of LS AC 1-1 c) and LS AC 1-3 f) samples.

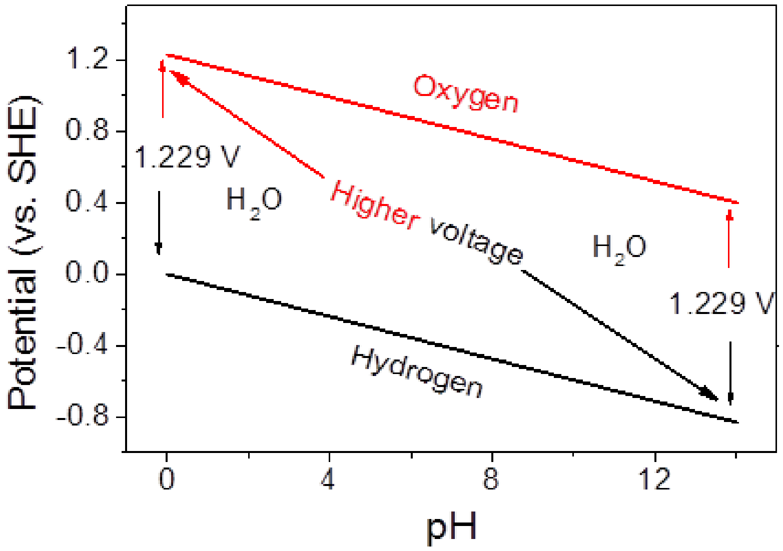

Figure S4. Change of stability potentials of hydrogen and oxygen with pH.

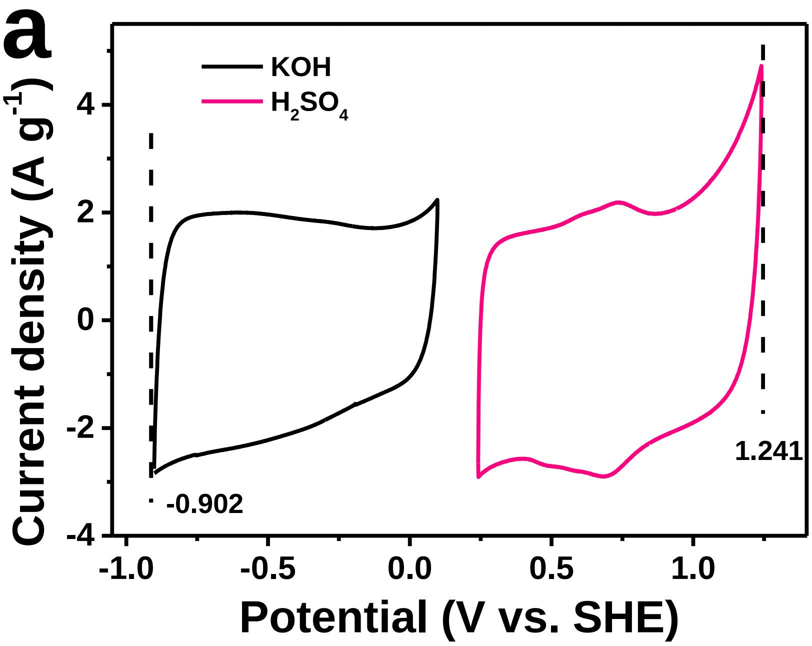

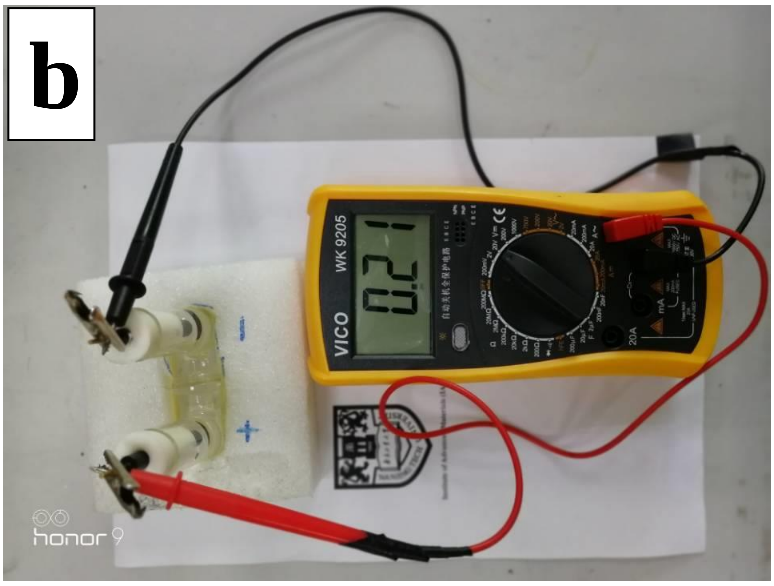

Figure S5. a) CV curves (vs. SHE) of LS AC at 10 mV s^{-1} in 2 M KOH and 1 M H_2SO_4 aqueous solutions. b) The potential difference of two electrolytes in the alkaline-acidic system (about 0.21 V).

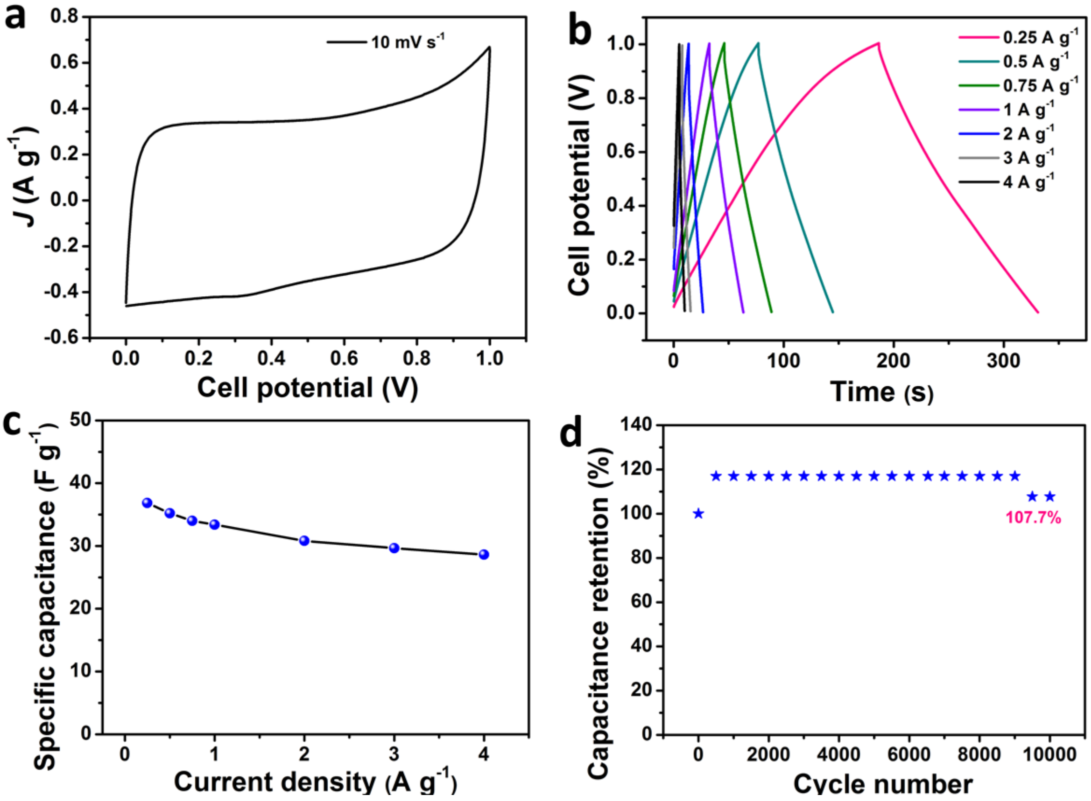

Figure S6. a) CV curve at 10 mV s^{-1} and b) GCD profiles at different current densities of LS AC-based supercapacitor in 2 M KOH aqueous electrolyte solution. c) Specific capacitance vs. current density of LS AC-based supercapacitor. e) Cycling stability of LS AC-based supercapacitor at 3 A g^{-1} over 10000 cycles.

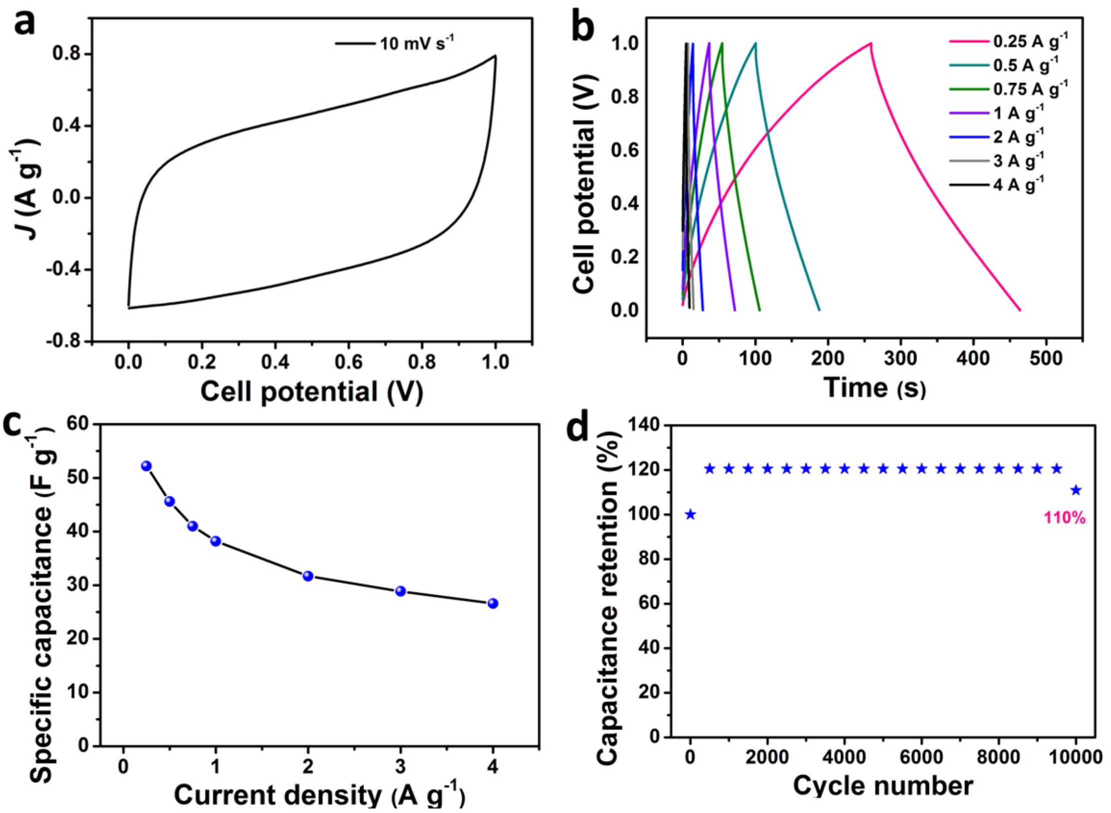

Figure S7. a) CV curve at 10 mV s^{-1} and b) GCD profiles at different current densities of LS AC-based supercapacitor in 1 M H_2SO_4 aqueous electrolyte solution. c) Specific capacitance vs. current density of LS AC-based supercapacitor. e) Cycling stability of LS AC-based supercapacitor at 3 A g^{-1} over 10000 cycles.

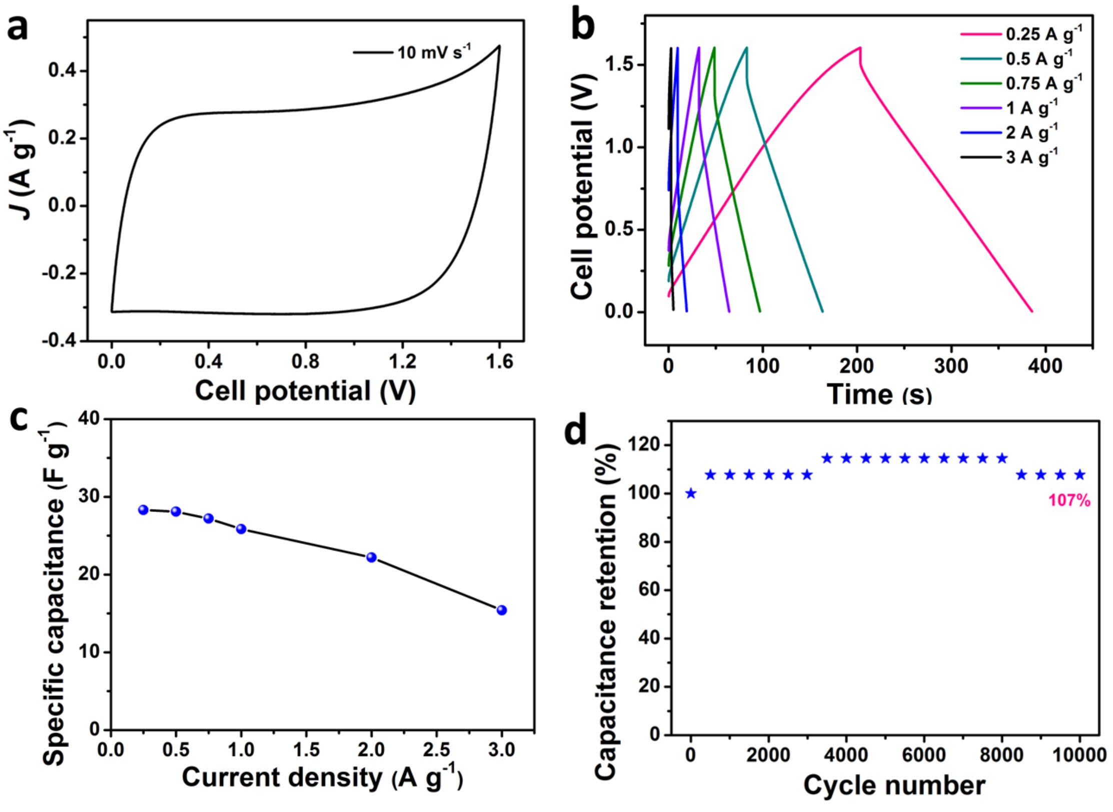

Figure S8. a) CV curve at 10 mV s^{-1} and b) GCD profiles at different current densities of LS AC-based supercapacitor in 1 M Li_2SO_4 aqueous electrolyte solution. c) Specific capacitance vs. current density of LS AC-based supercapacitor. e) Cycling stability of LS AC-based supercapacitor at 3 A g^{-1} over 10000 cycles.

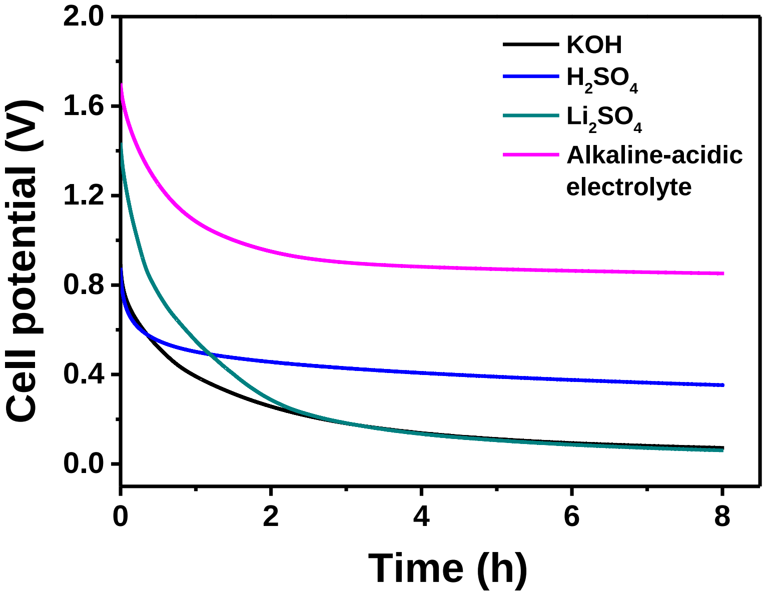

Figure S9. Open circuit voltage curve showing self-discharge behavior of LS AC-based supercapacitors in 2 M KOH, 1 M H_2SO_4, 1M Li_2SO_4, and alkaline-acidic electrolytes.

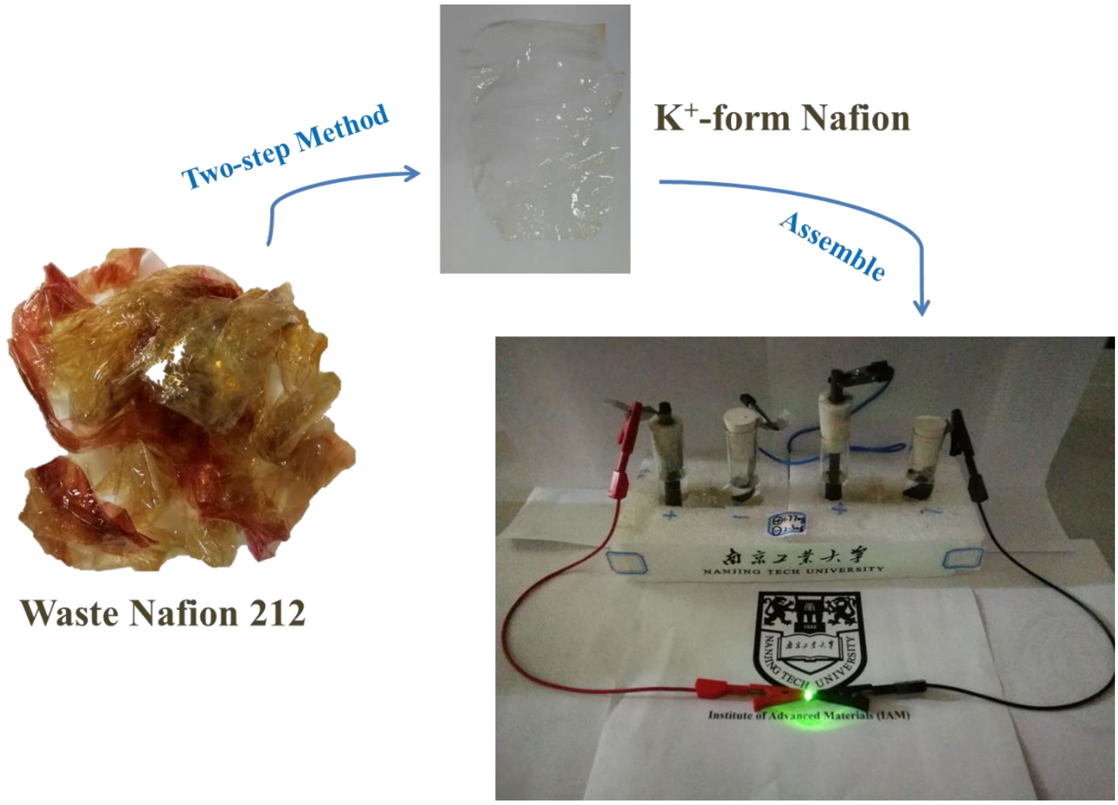

Figure S10. Construction of alkaline-acidic electrolyte and optical image of two simple cell lighting up a green LED (2.4 V).
